# Effects of CPAP on nitrate and norepinephrine levels in severe and mild-moderate sleep apnea

**DOI:** 10.1186/1471-2466-13-13

**Published:** 2013-03-13

**Authors:** Paula Pinto, Cristina Bárbara, Joseph M Montserrat, Rita S Patarrão, Maria P Guarino, Miguel M Carmo, Maria P Macedo, Cristina Martinho, Rita Dias, Maria JM Gomes

**Affiliations:** 1 Serviço de Pneumologia II, Centro Hospitalar Lisboa Norte-Hospital Pulido Valente, Lisbon, Portugal; 2 Faculdade de Ciências Médicas, Universidade Nova de Lisboa, Lisboa, Portugal; 3 Centro de Estudos de Doenças Crónicas, Lisbon, Portugal; 4 Hospital Clinic. CIBERES. IDIBAPS, Barcelona, Spain

## Abstract

**Background:**

Reduced plasma nitrate (NO_x_) levels and increased urinary norepinephrine (U-NE) levels have been described in severe obstructive sleep apnea (OSA), and are reverted by continuous positive airway pressure (CPAP). The effect of CPAP on these biomarkers in mild-moderate OSA is not well understood.

The aim of this study was to compare NO_x_ and U-NE levels and blood pressure (BP) between male patients with mild-moderate and severe OSA and determine the impact of 1 month of CPAP therapy on these parameters.

**Methods:**

We undertook a prospective study of 67 consecutive OSA patients (36 mild-moderate, 31 severe). Measurements of plasma NO_x_ at 11 pm, 4 am and 7 am, 24-h U-NE and ambulatory BP were obtained at baseline and after 1 month of CPAP.

**Results:**

At baseline, NO_x_ levels showed a significant decrease during the night in both groups (p < 0.001). U-NE level and BP were significantly higher in the severe OSA group. After 1 month of CPAP, there was a significant increase in NO_x_ levels and a reduction in U-NE level and BP only in patients with severe OSA.

**Conclusions:**

One month of CPAP results in significant improvements in NO_x_ levels, 24-h U-NE level and BP in patients with severe OSA, but not in patients with mild-moderate OSA.

**Trial registration:**

ClinicalTrials.gov: http://NCT01769807

## Background

Obstructive sleep apnea (OSA) is an increasingly recognized health issue. Considerable evidence supports an independent association between OSA and cardiovascular disease, which is particularly strong for systemic arterial hypertension [1-3]. The pathogenesis of this association is likely to be multifactorial, involving a diverse range of mechanisms including increased sympathetic activity, systemic inflammation, endothelial dysfunction, oxidative stress and metabolic dysregulation [4,5].

Continuous positive airway pressure (CPAP) decreases daytime somnolence and prevents cardiovascular complications in patients with severe OSA [6,7]. However, there is no consensus on the cardiovascular benefits of CPAP therapy in mild-moderate patients. The impact of mild forms of obstructive sleep apnea and their treatment on cardiovascular outcomes remains controversial. The observational study by Buchner et al. [8] showed increased cardiovascular morbidity in mild-moderate OSA patients and demonstrated that OSA treatment improved cardiovascular outcome. They found that cardiovascular events were more frequent in untreated mild-moderate OSA patients, and that OSA treatment was associated with a significant reduction (64%) in cardiovascular risk, independent of age, gender and pre-existing cardiovascular comorbidities.

Reduced circulating nitrate (NO_x_) levels, increased urinary norepinephrine (U-NE) levels and increased blood pressure (BP) have been described in patients with severe OSA, and are reverted by CPAP [9-17]. The effect of CPAP on these parameters has, however, not been studied in mild-moderate OSA. Previous studies evaluating NO_x_ deficiency and sympathetic dysfunction have mainly focused on patients with severe OSA.

To address this issue, we designed a prospective study that sought to: 1) compare NO_x_ and U-NE levels between male patients with mild-moderate and severe OSA; 2) compare BP values between these patient groups, and 3) determine whether CPAP therapy improves NO_x_ deficiency, sympathetic dysfunction and BP in these patients.

## Methods

### Study population

All patients were recruited from our institution’s sleep laboratory. OSA was diagnosed on the basis of symptoms and a respiratory disturbance index (RDI) showing more than 5 respiratory events/h on overnight polysomnography. We recruited 67 consecutive male patients with OSA: 36 with mild-moderate OSA and 31 with severe OSA.

Patients were excluded if they met any of the following criteria: 1) current smoker; 2) respiratory or cardiac disease (except for arterial hypertension); 3) renal, hepatic or psychiatric disorder; 4) diabetes mellitus or dyslipidemia; 5) rhinitis, sinusitis or acute illness; 6) daytime hypoxemia or hypercapnia; 7) therapy with oral nitrates, angiotensin-converting enzyme inhibitors, beta-blockers, statins or non-steroidal anti-inflammatory drugs; 8) presence of central respiratory events on polysomnography; 9) previous CPAP therapy or uvulopalatopharyngoplasty. One patient from each group was also excluded because of failure to comply with CPAP.

Patients were assessed for obstructive/restrictive lung disease by a respiratory physician based on clinical evaluation, pulmonary function testing, arterial blood gas measurement and chest radiography, and for cardiac disease by a cardiologist based on history, physical examination, electrocardiography and echocardiography.

As recommended for the measurement of NO_x_ levels, all subjects were instructed to ingest a low nitrate/nitrite diet [18]. They were also advised to avoid exercise on urine collection days.

The study protocol was approved by Centro Hospitalar Lisboa Norte’s Ethics Committee and all subjects gave written informed consent. The study was performed in accordance with the guidelines in the current revision of the Declaration of Helsinki.

### Study design

We performed a single-center, prospective study of OSA patients. Data were collected in all subjects at baseline and after 1 month of CPAP. These data included full poly-somnography, ambulatory blood pressure monitoring (ABPM), fasting venous blood collection at 11 pm, 4 am and 7 am for NOx levels and 24-h urine collection for U-NE. Epworth sleepiness scale was administered at all visits.

### Polysomnography

All subjects underwent full polysomnography (Embla S7000, Embla, USA). Sleep stages were manually scored according to the criteria of Rechtschaffen and Kales [19]. Respiratory events were classified according to the recent scoring recommendations [20]. Apnea was defined as a reduction of >90% in oronasal airflow lasting ≥10 seconds. Hypopnea was defined as a decrease of >30% in oronasal airflow lasting ≥10 seconds and associated with ≥4% desaturation from the pre-event baseline. Respiratory effort related arousals were defined as a sequence of breaths lasting at least 10 seconds characterized by flattening of the nasal pressure waveform and leading to an arousal from sleep. OSA was defined when RDI was ≥5 events/h (mild-moderate was defined as RDI <30 and severe as RDI ≥30).

### CPAP therapy

One week after the initial visit, an automated pressure setting device (AutoSet Spirit, ResMed, Australia) was given to all patients with predefined settings (minimal pressure: 4 cmH_2_O; maximal pressure: 16 cmH_2_O) and they were instructed to use this for 1 month. CPAP adaptation was performed by a sleep technician and patients underwent an educational program for appropriate use of the equipment. Patients were considered compliant with CPAP if they used it for ≥4.5 h/night. Follow-up program consisted of weekly phone calls and immediate correction of adverse effects with optimization of CPAP settings, if necessary.

### Ambulatory blood pressure monitoring

All patients underwent ABPM with a non-invasive portable validated recorder (Spacelab 90207, SpaceLabs Medical, Redmond, WA, USA) at baseline and after 1 month of CPAP. A BP cuff was fitted on the non-dominant arm of the patient for 24 h. BP was recorded every 20 min during the day and every 30 min during the night. A dipping pattern was defined as a reduction in the average systolic BP at night of >10% compared with daytime values. Based on ABPM, hypertension was classified according to the recent guidelines for the management of arterial hypertension [21]. For ethical reasons, anti-hypertensive treatment was not withdrawn during the study.

### Blood sample collection and measurement of NO_x_

An intravenous catheter was inserted into an antecubital vein and was kept patent by a continuous saline drip.

Plasma NO_x_ levels were determined by the chemiluminescence technique, using a Sievers 280 NO Analyzer (Sievers Instruments) as previously described [22-24].

### Urinary catecholamines analysis

Sympathetic nervous system activity was estimated by 24-h U-NE levels using high-performance liquid chromatography as previously described [25,26].

### Statistical analysis

Results are presented as mean ± SD or as percentages. Comparisons between patients with mild-moderate and severe OSA were performed using the non-paired *t*-test or the Mann–Whitney *U* test when comparing mean values between groups at baseline, and the paired *t*-test when comparing mean values before and after CPAP treatment. Multivariate ANOVA analysis was performed to assess changes in plasma NO_x_ levels during the night and to compare these values before and after CPAP.

Subjects were stratified by BMI > 30 and BMI < 30 kg/m2 to assess the effects of obesity on NOx and U-NE levels. Association between categorical variables was evaluated by the chi-square test or the Fisher Exact test. Bivariate relationships between variables were determined by Pearson’s or Spearman’s correlation. A p value of <0.05 was considered statistically significant.

Statistical analysis was performed using the Statistical Package for Social Sciences (SPSS) version 17.0 for Windows.

## Results

Table 1 shows characteristics of the study population at baseline and after 1 month of CPAP. Sixty-seven consecutive OSA patients were studied: 36 patients with mild-moderate OSA and 31 patients with severe OSA.

**Table 1:** Characteristics of the study population at baseline and after 1 month of CPAP

	**Mild/moderate OSA patients (n = 36)**		**Severe OSA patients (n = 31)**	
	**Baseline**	**Post CPAP**	**Baseline**	**Post CPAP**
Age (yrs)	48 ± 10.4	NA	51 ± 6.3	NA
Body mass index (Kg/m^2^)	29 ± 3.8*	30 ± 3.8	35 ± 5.1*	35 ± 5.0
Epworth score	11 ± 6.2^#^	3.1 ± 2.5	12 ± 6.3 ^#^	4.2 ± 3.2
RDI (n/h)	16 ± 6.8*	^#^ 3 ± 2.5	66 ± 24.2^#^	4 ± 2.3
Mean SaO_2_ (%)	95 ± 1.3*	^#^ 96 ± 1.2	91 ± 4.8^#^	95 ± 1.2
Minimum SaO_2_ (%)	86 ± 4.8*	^#^ 93 ± 2.1	71 ± 9.9^#^	93 ± 2.2
SaO_2_ < 90% (% of TST)	1 ± 3.2*	^#^ 0 ± 0.2	27 ± 26.6^#^	0 ± 0.1
ODI (n/h)	10 ± 9.5*	^#^ 0 ± 0.2	52 ± 20.5^#^	0 ± 0.2
Arousal index (n/h)	23 ± 8.1*	^#^ 16 ± 9.2	53 ± 18^#^	14 ± 7
Compliance (h/night)	NA	6.3 ± 1.3	NA	6.6 ± 0.9
95^th^ percentile CPAP pressure (cmH_2_O)	NA	11.5 ± 1.8	NA	13.2 ± 1.5
24h MAP (mmHg)	91 ± 6.8*	92 ± 6.9	97 ± 8.1^#^	92 ± 9
Daytime BP (mmHg)				
Systolic	127 ± 8.3*	127 ± 8.8	134 ± 10.9^#^	130 ± 10.1
Diastolic	81 ± 6.2*	70 ± 7.5	85 ± 5.8 ^#^	80 ± 6.2
Nighttime BP (mmHg)				
Systolic	114 ± 7.9*	116 ± 10.3	125 ± 14.2^#^	119 ± 12.9
Diastolic	70 ± 7.6*	70 ± 7.5	76 ± 9.3^#^	73 ± 8.9
No_x_ 11pm (μm)	34.3 ± 21.8^+^	41.3 ± 22.2	34.5 ± 21.9 ^&^	41.4 ± 19.8
No_x_ 4am (μm)	29.4 ± 15.4^+^	37.7 ± 21.1	27.2 ± 14.4 ^&^	37.4 ± 17.9
No_x_ 7am (μm)	26.4 ± 14.1^+^	36.6 ± 19.5	24.3 ± 16.8 ^&^	35.2 ± 16.7
ΔNO_X_ (%)	16.5 ± 18.5^+^	9.3 ± 16.3	27.6 ± 20.1 ^&^	0.79 ± 52.2
U-NE (μg/24h)	48.5 ± 19.9^*^	47 ± 21.3	73.9 ± 30.1^#^	55.4 ± 21.8

Values shown as mean ± SD. RDI, respiratory disturbance index; SaO_2,_ arterial oxygen saturation; TST, total sleep time; ODI, oxygen desaturation index; MAP, mean arterial pressure; BP, blood pressure; NO_X_, nitrate levels; ΔNO_X_, percentage decrease in NO_x_ during the night; U-NE, urinary norepinephrine; NA, Not applicable.

*: p < 0.05 comparison of baseline values between patients with severe and mild-moderate OSA (unpaired *t*-test).

#: p < 0.05 comparison between pre- and post-CPAP values (paired *t*-test).

^+^ p < 0.05 comparison between different time points and between pre- and post-CPAP for patients with mild-moderate OSA (multivariate ANOVA analysis).

^&^ p < 0.05 comparison between different time points and between pre- and post-CPAP for patients with severe OSA (multivariate ANOVA analysis).

Patients with severe OSA had a significantly higher body mass index (BMI) than patients with mild-moderate OSA (p < 0.05). Also, those patients had more severe oxygen desaturation than mild-moderate OSA (p < 0.05).

### Before CPAP

At baseline, 61% of patients with severe OSA and 53% of patients with mild-moderate OSA had excessive daytime sleepiness with an Epworth sleepiness scale (ESS) score >10 (p = 0.80). After CPAP, excessive daytime sleepiness was completely reversed in all patients with severe OSA and in 94% of patients with mild-moderate OSA (2 mild-moderate patients still had an ESS score >10 after CPAP; p = 0.13).

Patients with severe OSA had significantly higher BP values than patients with mild-moderate OSA (Table 1). Before CPAP, 83% of patients with severe OSA compared with 69% of patients with mild-moderate OSA had arterial hypertension (p = 0.93), however only 31% of patients with severe OSA and 29% with mild-moderate OSA were treated for hypertension. All treated hypertensive patients with mild-moderate OSA had controlled BP levels at baseline and 39% of them showed a non-dipper nocturnal pressure pattern. In contrast, 55% of patients with severe OSA showed a non-dipper pattern (p > 0.05). The hypertensive medications were: calcium antagonists, diuretics and angiotensin II receptor antagonists.

**Figure 1:**
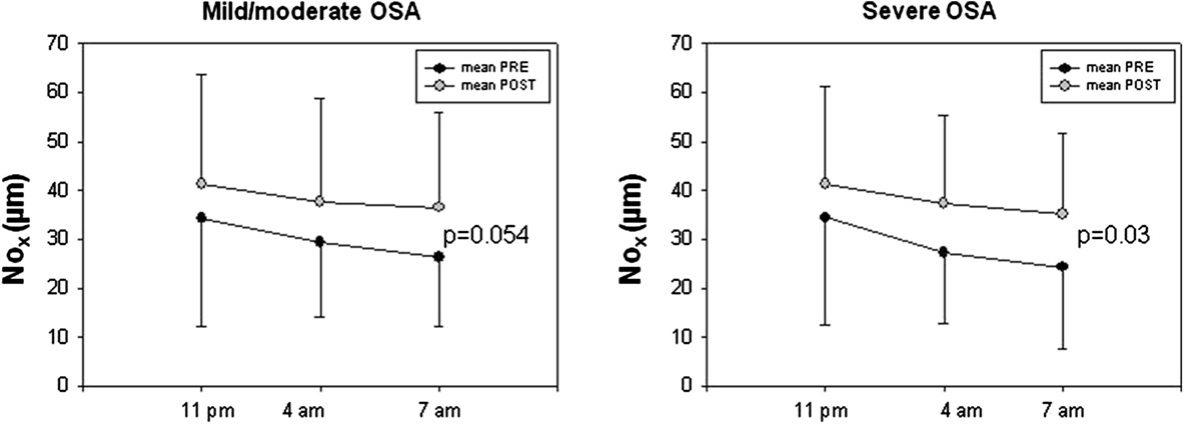
NOx levels at different time points before and after 1 month of CPAP in patients with mild-moderate and severe OSA.

Before CPAP, there were no differences in NO_x_ levels between patients with mild-moderate and severe OSA for all times measured. NO_x_ levels showed a significant decrease during the night in both groups (p < 0.001) (Table 1, Figure 1). The percentage of NO_x_ reduction during the night (ΔNO_x_) was significantly higher in patients with severe OSA than in patients with mild-moderate OSA (27.6 ± 20.1% vs 16.5 ± 18.5%, p < 0.05).

**Figure 2:**
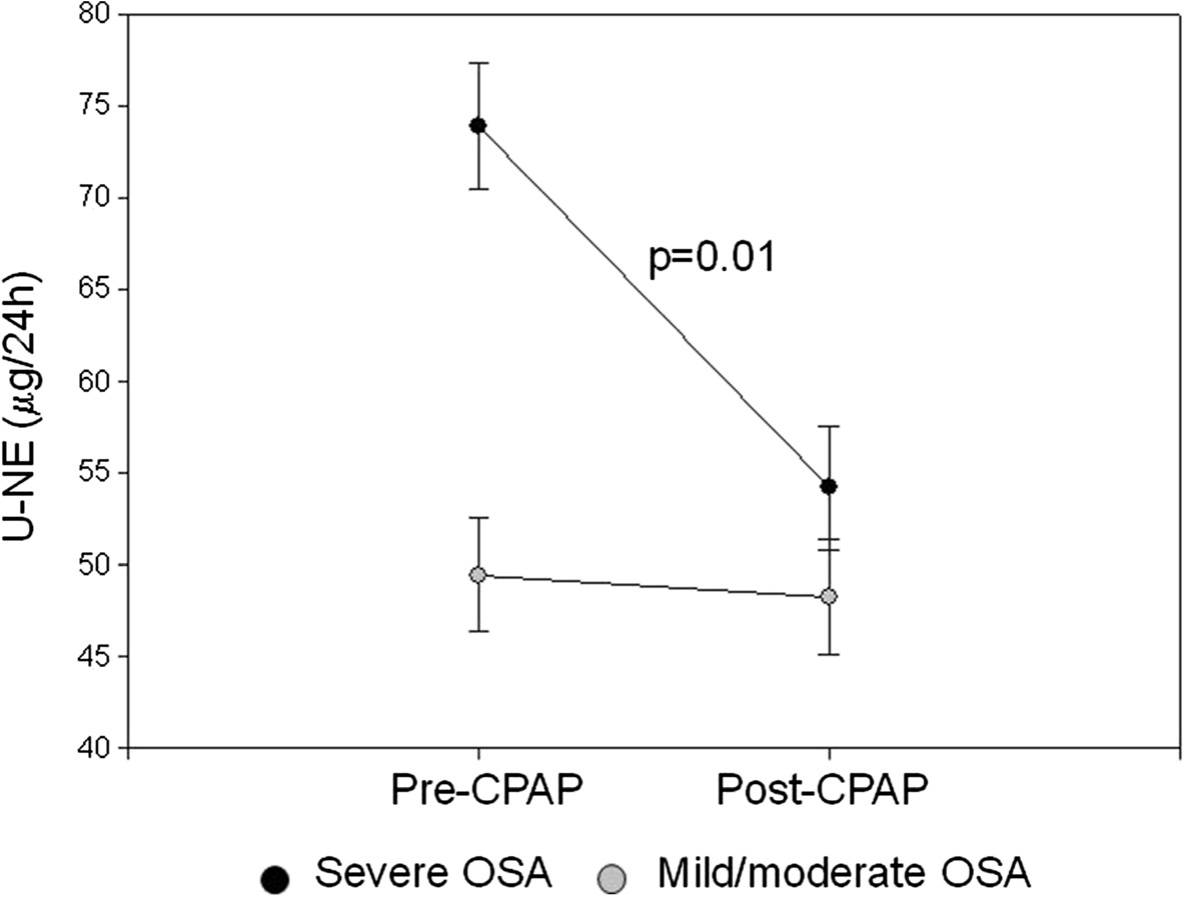
**U**-**NE levels before and after 1 month of CPAP in patients with mild**-**moderate and severe OSA.**

24-h U-NE levels were significantly higher in patients with severe OSA than in patients with mild-moderate OSA (73.9 ± 30.1 vs 48.5 ± 19.91 μg/24h, p < 0.05) (Table 1, Figure 2). There was a significant cor-relation between U-NE levels and the sleep parameters RDI (r = 0.63; p < 0.001), mean SaO_2_ (r = −0,47; p < 0.001), minimum SaO_2_ (r = −0.55; p < 0.001) and arousal index (r = 0.59; p < 0.001).

The study population was also stratified by BMI > 30 and < 30 kg/m2 and there was no difference between obese and non obese subjects in NOx and U-NE levels.

### After CPAP

All patients who completed the study were CPAP compliant. Compliance was similar in the mild-moderate and severe groups (6.3 ± 1.3 vs 6.6 ± 0.9 h/night; p > 0.05). 95^th^ percentile CPAP pressure was 11.5 ± 1.8 cm H_2_O in patients with mild-moderate OSA and 13.2 ± 1.5 cm H_2_O in patients with severe OSA (p < 0.001). BMI did not change significantly during the study period. RDI and SaO_2_ indexes normalized in both patient groups.

After CPAP, both groups had a rise in NO_x_, although in patients with mild-moderate OSA rise in NOx did not reach full statistical significance (p = 0.054; Figure 1, Table 1). Comparison of the percentage in NO_x_ level reduction during the night (ΔNO_x_) pre- and post-CPAP showed a significant decrease only in patients with severe OSA (27.6 ± 20.1% vs 0.79 ± 52.2%; p < 0.05).

Evaluation of sympathetic activity showed a significant reduction in 24-h U-NE excretion after CPAP only in patients with severe OSA (73.9 ± 30.1 to 55.4 ± 21.8 μg/24 h; p < 0.05) (Table 1, Figure 2). Patients with severe OSA had higher post-CPAP U-NE levels than patients with mild-moderate OSA, however the difference was not statistically significant.

After 1 month of CPAP, patients with severe OSA had a significant decrease in 24-h MAP (p < 0.05), daytime systolic BP (p < 0.05) and diastolic BP (p < 0.05), and also night-time systolic BP (p < 0.05) and diastolic BP (p < 0.05). CPAP did not change BP in patients with mild-moderate OSA. However, it is worth noting that final BP values of patients with severe OSA were similar to BP levels of patients with mild-moderate OSA. Normal circadian dipper pattern was not restored in non-dipper patients after 1 month of CPAP.

## Discussion

Our study showed that there was a significant decrease in plasma NO_x_ levels during the night in patients with both severe and mild-moderate OSA before CPAP, with the percentage reduction being higher in patients with severe OSA. After 1 month of CPAP, there was a significant increase in NO_x_ levels in patients with severe OSA and a non-significant increase in patients with mild-moderate OSA. We demonstrated that patients with severe OSA had higher baseline U-NE levels than patients with mild-moderate OSA. After 1 month of CPAP, only patients with severe OSA had significantly decreased U-NE levels compared with pre-CPAP levels. We also found that 1 month of CPAP treatment reduced BP values only in patients with severe OSA.

The main strengths of our study were the experimental design (determination of NO_x_ levels during the night) and the comparison between two groups of patients with different OSA severity.

### NO_x_ deficiency

Diet is a contributor to NO_x_ levels [18] which has not been addressed in previous studies [9,12]. In our study all subjects were instructed to ingest a low nitrate/nitrite diet.

Previous studies have demonstrated that NO_x_ production is reduced in severe OSA [9,10,12]. We further evaluated different severity OSA patient groups through the night. We demonstrated a significant decrease in NO_x_ levels during the night in patients with mild-moderate and severe OSA, with a higher percentage reduction in NO_x_ levels in patients with severe OSA. Lavie et al. also reported a decrease in NO_x_ levels during the night in eight patients with severe OSA [11].

There is some evidence that hypoxemia associated with apnea could lead to decreased nitric oxide production. As oxygen is a cosubstrate of nitric oxide (NO) synthase (NOS), OSA related desaturations might result in depressed synthesis of NO. Night-time hypoxia might suppress the transcription of the endothelial NOS gene and the stability of its mRNA, as suggested by cell culture experiments performed under hypoxic conditions [27]. It has been demonstrated that NOS inhibitors are increased in OSA and might contribute to lowered NO levels [28].It is also possible that free oxygen radicals generated by circulating neutrophils under conditions of hypoxia-reoxygenation in untreated OSA might cause an exaggerated destruction of NO [29]. These mechanisms were not evaluated in the present study.

Previous studies have shown that in patients with severe OSA, CPAP therapy to abolish sleep apnea results in increased NO_x_ levels, suggesting that whatever mechanism causes the suppression of NO synthesis or release is acute and promptly reversible with the reversal of sleep apnea [9-12]. Our study confirmed these results, but did not show an increase in NO_x_ levels in mild-moderate patients. However, this finding could be related to low statistical power. Indeed, a higher sample would probably reach statistical significance.

### Sympathetic dysfunction

Previous studies have demonstrated high U-NE levels in severe OSA patients [13-17]. Our findings showed significantly higher 24-h U-NE levels in patients with severe OSA compared with patients with mild-moderate OSA. U-NE levels correlated with RDI, overnight oxygen desaturation and arousal index, suggesting that hypoxemia and sleep fragmentation increased sympathetic activity.

It has also been shown that CPAP reduces U-NE levels in patients with severe OSA [13,15,17]. Our study confirmed these results for patients with severe OSA and demonstrated that there was no change in U-NE levels following CPAP in patients with mild-moderate OSA. The mechanisms explaining this observation are unclear, but we propose that in patients with mild-moderate OSA, the overnight oxygen desaturation and the arousal index related to respiratory events were not sufficient to activate the sympathetic nervous system and thereby modify U-NE levels. After 1 month of CPAP, U-NE levels of patients with severe OSA were not significantly different from those with mild-moderate OSA.

### Blood pressure

Previous studies have demonstrated that CPAP reduces BP in patients with severe OSA [30-32]. This study also found that both systolic and diastolic BP was reduced in daytime and night-time measurements in patients with severe OSA. In patients with mild-moderate OSA, no change in BP values was found; however these patients already had their BP controlled within the normal range at baseline. Alonso-Fernández [12] also found that normotensive patients did not change their BP values after CPAP.

The meta-analysis by Haentjens [33] showed that greater CPAP related reductions in ambulatory BP were observed in patients with more severe OSA and a better nightly use of CPAP.

In our study, differences in the effects of CPAP on BP between patients with mild-moderate OSA and patients with severe OSA could not be explained by differences in compliance rates, as compliance was similar between groups. The high rate of CPAP compliance might be explained by a very comprehensive follow-up program with weekly phone calls and immediate correction of adverse effects.

The non-reversal of the circadian BP pattern in our patients might be due to the short period of CPAP treatment. In the study by Martinez Garcia [30], normalization of the nocturnal BP pattern occurred after three months of CPAP.

In this study, we found that 1 month of CPAP therapy did not change NO_x_ levels, 24-h U-NE levels or BP values in patients with mild-moderate OSA. The absence of response of one clinical parameter (BP) and two biological markers (NO_x_ and U-NE) to CPAP therapy could suggest that physiological effects of mild-moderate OSA may not be disruptive enough to stimulate a rise in U-NE and BP, as does severe OSA. It could also be that a 1 month of CPAP was not enough to detect differences between the groups. Also, CPAP might not have a protective cardiovascular effect in less severe forms of OSA. Indeed, mortality follow-up of the Wisconsin Sleep Cohort, including 20,963 subjects, indicated that only patients with severe OSA had a significantly increased cardiovascular mortality risk [34].

Limitations:

Some limitations should be addressed in our study, namely a low sample population could compromise statistical power. Also, our data are unable to generalize to female, as we did not include women.

As apnea hypopnea index is more often referred in the literature, using RDI could difficult the comparison between studies. Also, the maintenance of antihypertensive therapy could have confounded results. The short time of CPAP treatment is another possible limitation, as a longer study period may have produced different results. In the study by Martinez Garcia et al. [30], normalization of the nocturnal BP pattern occurred after three months of CPAP.

As our results may have implications for the CPAP treatment of patients with mild-moderate OSA, it is important to undertake a larger controlled study with a longer follow-up period.

## Conclusions

Our study demonstrated that 1 month of CPAP treatment significantly increases plasma NO_x_ levels and reduces 24-h U-NE levels and BP in patients with severe OSA, but did not demonstrate a significant change in these values in patients with mild-moderate OSA.

## Competing interests

The authors declare that they have no competing interests.

## Authors’ contributions

PP participated in study conception, design and data acquisition, and drafting of the manuscript. CB participated in study conception and design and drafting of the manuscript. JM participated in drafting of the manuscript. RP and MG performed the NO_x_ analysis. MC and MM participated in study design. CM and AD performed the polysomnographies. MG participated in study design. All authors read and approved the final manuscript.

## Pre-publication history

The pre-publication history for this paper can be accessed here:

http://www.biomedcentral.com/1471-2466/13/13/prepub

## References

[B1] Shahar E Whitney CW Redline S Lee ET Newman AB Nieto FJ O’connor GT Boland LL Schwartz JE Samet JM Sleep-disordered breathing and cardiovascular disease: cross-sectional results of the Sleep Heart Health Study Am J Respir Crit Care Med 2001 163 19 25 11208620 10.1164/ajrccm.163.1.2001008

[B2] Peppard PE Young T Palta M Skatrud J Prospective study of the association between sleep-disordered breathing and hypertension N Engl J Med 2000 342 1378 1384 10.1056/NEJM200005113421901 10805822

[B3] Nieto FJ Young TB Lind BK Shahar E Samet JM Redline S D’Agostino RB Newman AB Lebowitz MD Pickering TG for the Sleep Heart Health Study Association of sleep-disordered breathing, sleep apnea and hypertension in a large community-based study JAMA 2000 283 1829 1836 10.1001/jama.283.14.1829 10770144

[B4] McNicholas WT Bonsignore MR and the Management Committee of EU COST ACTION B26 Sleep apnoea as an independent risk factor for cardiovascular disease: current evidence, basic mechanisms and research priorities Eur Respir J 2007 29 156 178 17197482 10.1183/09031936.00027406

[B5] Bradley TD Floras JS Obstructive sleep apnea and its cardiovascular consequences Lancet 2009 373 82 93 10.1016/S0140-6736(08)61622-0 19101028

[B6] Loube DI Gay PC Strohl KP Pack AI White DP Collop NA Indications for positive airway pressure treatment of adult obstructive sleep apnea patients Chest 1999 115 863 866 10.1378/chest.115.3.863 10084504

[B7] Kushida CA Littner MR Hirshkowitz M Practice parameters for the use of continuous and bilevel positive airway pressure devices to treat adult patients with sleep-related breathing disorders Sleep 2006 29 375 380 16553024 10.1093/sleep/29.3.375

[B8] Buchner NJ Sanner BM Borgel J Rump LC CPAP treatment of mild to moderate obstructive sleep apnea reduces cardiovascular risk Am J Respir Crit Care Med 2007 176 1274 1280 10.1164/rccm.200611-1588OC 17673692

[B9] Ip MSM Lam B Chan L Zheng L Tsang KWT Fng PCW Lam W Circulating nitric oxide is suppressed in obstructive sleep apnea and is reversed by nasal continuous positive airway pressure Am J Respir Crit Care Med 2000 162 2166 2171 11112132 10.1164/ajrccm.162.6.2002126

[B10] Schulz R Schmidt D Blum A Lopes-Ribeiro X Lucke C Mayer K Olschewski H Seeger W Grimminger F Decreased plasma levels of nitric oxide derivatives in obstructive sleep apnoea: response to CPAP therapy Thorax 2000 55 1046 1051 10.1136/thorax.55.12.1046 11083891 PMC1745665

[B11] Lavie L Hefetz A Luboshitzky R Lavie P Plasma levels of nitric oxide and L-arginine in sleep apnea patients. Effects of nCPAP treatment J Mol Neurosci 2003 21 57 63 10.1385/JMN:21:1:57 14500996

[B12] Alonso-Fernández A Garcia-Rio F Arias MA Hernanz A Peña M Pierola J Barceló A López-Collazo E Garcia AA Effects of CPAP upon oxidative stress and nitrate efficiency in sleep apnoea A randomized trial. Thorax 2009 64 581 586 10.1136/thx.2008.100537 19074930

[B13] Sukegawa M Noda A Sugiura T Nakata S Yoshizaki S Soga T Yasuda Y Iwayama N Nakai S Koike Y Assessment of continuous positive airway pressure treatment in obstructive sleep apnea syndrome using 24-hour urinary catecholamines Clin Cardiol 2005 28 519 522 10.1002/clc.4960281106 16450795 PMC6653944

[B14] Elmasry A Lindberg E Hedner J Janson C Boman G Obstructive sleep anoea and urine catecholamines in hypertensive males: a population-based study Eur Respir J 2002 19 511 517 10.1183/09031936.02.00106402 11936531

[B15] Hedner J Darpo B Ejnell H Carlson J Caidahl K Reduction in sympathetic activity after long-term CPAP treatment in sleep apnoea: cardiovascular implications Eur Respir J 1995 8 222 229 10.1183/09031936.95.08020222 7758555

[B16] Marrone O Riccobono L Salvaggio A Mirabella A Bonanno A Bonsignore MR Catecholamines and blood pressure in obstructive sleep apnea syndrome Chest 1993 103 722 727 10.1378/chest.103.3.722 8449058

[B17] Ziegler MG Mills PJ Loredo JS Ancoli-Israel S Dimsdale JE Effect of continuous positive airway pressure and placebo treatment on sympathetic nervous activity in patients with obstructive sleep apnea Chest 2001 120 887 893 10.1378/chest.120.3.887 11555525

[B18] Wang J Brown MA Tam SH Chan MC Whitworth JA Effects of diet on measurement of nitric oxide metabolites Clinical and Experimental Pharmacology and Physiology 1997 24 418 420 10.1111/j.1440-1681.1997.tb01212.x 9171946

[B19] Rechtschaffen A Kales A A manual of standardized terminology, techniques and scoring system for sleep stages of human subjects 1968 Education and Welfare Public Health Service – NIH/NIND: US Department of Health

[B20] Iber CI Ancoli-Israel S Chesson L Quan Sfor the American Academy of Sleep Medicine 2007 Rules, terminology and technical specifications: The AASM Manual for the Scoring of Sleep and Associated Events

[B21] Mancia G Backer G Dominiczack A Cifkova R Fagard R Germano G Grassi G Heagerty A Kjeldsen S Laurent S Narkiewicz K Ruilope L Rynkiewicz A Schmieder R Boudier H Zanchetti A Guidelines for the management of arterial hypertension. The task force for the management of arterial hypertension of the European Society of Hypertension (ESH) ad of the European Society of Cardiology (ESC) Journal of Hypertension 2007 25 1105 1187 10.1097/HJH.0b013e3281fc975a 17563527

[B22] Yang F Troncy E Francoeur M Vinet B Vinay P Czaika G Blaise G Effects of reducing reagents and temperature on conversion of nitrite and nitrate to nitric oxide and detection of NO by chemiluminescence Clin Chem 1997 43 657 662 9105269

[B23] Cox RD Frank CW Determination of nitrate and nitrite in blood and urine by chemiluminescence J Anal Toxicol 1982 6 148 152 7109559 10.1093/jat/6.3.148

[B24] Braman RS Hendrix SA Nanogram nitrite and nitrate determination in environmental and biological materials by vanadium (III) reduction with chemiluminescence detection Anal Chem 1989 61 2715 2718 10.1021/ac00199a007 2619057

[B25] Macdonald IA Lake DM An improved technique for extracting catecholamines from body fluids J Eurosci Meth 1985 13 239 248 10.1016/0165-0270(85)90072-X 4010333

[B26] Laederach K Weidmann P Pasma and urinary catecholamines as related to renal function in man Kidney Inc 1987 31 107 111 10.1038/ki.1987.16 3560639

[B27] McQuillan LP Leung GK Marsden PA Hypoxia inhibits expression of eNOS via transcriptional and post-transcriptional mechanisms Am J Physiol 1994 267 1921 1927 10.1152/ajpheart.1994.267.5.H1921 7526714

[B28] Carlson J Hedner J Pettersson A Increased plasma concentration of ADMA, a naturally occurring nitric oxde synthesis inhibitor in OSA patients Am J Respir Crit Care Med 1997 155 A869

[B29] Schulz R Mahmoudi S Hattar K Enhanced release of superoxide from polymorphonuclear neutrophils in obstructive sleep apnea: impact of CPAP therapy Am J Respir Crit Care Med 2000 162 566 570 10934088 10.1164/ajrccm.162.2.9908091

[B30] Martinez-Garcia MA Gómez-Aldaravi R Soler-Cataluna JJ Martinez TG Bernácer-Alpera B Román-Sánchez P Positive effect of CPAP treatment on the control of difficult-to-treat hypertension Eur Respir J 2007 29 951 957 10.1183/09031936.00048606 17301092

[B31] Pepperell JC Ramdassing-Dow S Crosthwaite N Mllins R Jenkinson C Stradling JR Davies RJO Ambulatory blood pressure after therapeutic and subtherapeutic nasal continuous positive airway pressure for obstructive sleep apnoea: a randomised parallel trial Lancet 2002 359 204 210 10.1016/S0140-6736(02)07445-7 11812555

[B32] Faccenda JF Mackay TW Boon NA Douglas NJ Randomized placebo-controlled trial of continuous positive airway pressure on blood pressure in the sleep apnea-hypopnea syndrome Am J Respir Crit Care Med 2001 163 344 348 11179104 10.1164/ajrccm.163.2.2005037

[B33] Haentjens P Van Meerhaeghe A Moscariello A Weerdt S Poppe K Dupont A Velkeniers B The impact of continuous positive airway pressure on blood pressure in patients with obstructive sleep apnea syndrome. Evidence from a meta-analysis of plaebo-controlled randomized trials Arch Intern Med 2007 167 757 765 10.1001/archinte.167.8.757 17452537

[B34] Young T Finn L Peppard PE Szklo-Coxe M Austin D Nieto J Stubb R Hla KM Sleep disordered breathing and mortality: eighteen-year follow-up of the Wisconsin Sleep Cohort Sleep 2008 31 1071 1078 18714778 PMC2542952

